# Perception of dignity in older men and women in the early stages of dementia: a cross-sectional study

**DOI:** 10.1186/s12877-022-03362-3

**Published:** 2022-08-18

**Authors:** Lucie Klůzová Kráčmarová, Jitka Tomanová, Kristýna A. Černíková, Peter Tavel, Kateřina Langová, Peta Jane Greaves, Helena Kisvetrová

**Affiliations:** 1 Olomouc University Social Health Institute, Sts. Cyril and Methodius Faculty of Theology, Palacky University Olomouc, Olomouc, Czech Republic; 2 The Centre for Research and Science, Faculty of Health Sciences, Palacký University Olomouc, Olomouc, Czech Republic; 3 Department of Nursing, Midwifery and Health, Faculty of Health and Life Sciences, Northumbria University, Newcastle upon Tyne, UK

**Keywords:** Activities of daily living, Attitude to aging, Dementia, Depression, Dignity, Gender, Older adults, Pain, Visual impairment

## Abstract

**Background:**

Dementia is a serious problem in old age, that impacts an individual’s ability to function and may threaten personal dignity. Given the variable features of the illness and the diversity of life experiences, many factors may contribute to the perception of dignity by men and women with dementia. The purpose of the study was to explore the factors that contribute to dignity and its domains in men and women with dementia.

**Methods:**

This cross-sectional study involved 316 community-dwelling patients with early-stage dementia (aged ≥ 60) (PwD). We assessed the participants’ sociodemographic and social involvement characteristics, health-related variables (pain, depression, physical performance, visual and hearing impairments), attitude to aging, and self-sufficiency in the activities of daily living (ADL). These factors were investigated as independent variables for the perception of dignity and of its domains in men and women.

**Results:**

Multivariate regression analysis showed that PwD experienced minor dignity problems in the early stages of dementia. In both men and women higher rates of depression, negative attitudes to aging, and pain were associated with reductions in the perception of dignity. In men, but not in women visual impairment had a negative effect on overall dignity, and on the associated domains of ‘Loss of Autonomy’ and ‘Loss of Confidence’. In women, lowered self-sufficiency in ADL contributed to reduced self-perception of dignity and in the associated domains of ‘Loss of Purpose of Life’, ‘Loss of Autonomy’, and ‘Loss of Confidence’. Sociodemographic and social involvement characteristics, hearing impairment, and physical performance did not influence the participants’ self-perception of dignity.

**Conclusion:**

The results suggested that several common factors (depression, attitudes to aging, and pain) contribute to the perception of dignity in both men and women. Other factors, visual impairments in men, and self-sufficiency in ADL in women, appear to be more gender specific. These differences might relate to their specific gender roles and experiences. The self-perception of dignity in PwD can be helped by supporting the individual, to the extent that their illness allows, in maintaining activities that are important to their gender roles, and that preserve their gender identity.

**Trial registration:**

NCT04443621.

## Introduction

### Dignity in people with dementia

Dementia is a neurodegenerative disease characterized by progressive, irreversible, and (as yet) incurable cognitive decline. People with dementia (PwD) retain their positive personality traits and character, however, as the illness progresses, symptoms such as memory loss; speech impairment; disorientation; dependency in the activities of daily living (ADL); and self-neglect, are common [1]. In the early stage of dementia, patients are able to reflect on their disease. Awareness of a deteriorating condition and related symptoms increases the risk of depression, anxiety, and reduced quality of life [2]. It can also lead to a reduced perception of their dignity [3].

Dignity can be defined as a multidimensional construct that includes perception, knowledge, and emotions related to competence or respect [4]. It is a subjective experience of individuals’ own self-worth and self-esteem, as well as the respect and esteem that others show them [5, 6]. Nordenfelt [7] suggested that the dignity of identity is particularly crucial in the context of illness and old age. In older adults, frailty, dependence, sensory impairments, and cognitive decline tend to compromise dignity [8]. Based on these assumptions, it would be appropriate to address the area of dignity specifically for older adults with dementia. However, regarding dementia, dignity has most often been examined from the point of view of health professionals or other caregivers [9]. The limited number of studies that have focused on the issue from the point of view of the people with dementia (PwD) found that a threat to dignity existed, to varying degrees, in both PwD living in institional care [10, 11], and those living in their own home [3, 9, 12, 13].

Some of these studies have also suggested which variables might be related to the perceived dignity of PwD [10, 12]. Reduced self-sufficiency in ADL and increased dependence on caregivers were among the factors influencing the dignity of PwD living in nursing homes [10]. In community-dwelling PwD, dignity correlated positively with a higher degree of self-sufficiency in ADL, a lower level of depression, and better attitudes to aging [12]. Attitudes to aging are social constructs that are culturally and historically situated, and individually interpreted [14]. They relate to physical and social losses and gains in the past and present, and psychological growth, which can then be reflected in the sense of personal dignity [15]. In the study of Kisvetrová et al. [12], women with dementia associated aging with psychosocial loss (experience of loneliness), social exclusion, and the gradual worsening of physical self-sufficiency. How gender contributes to the domains of the Patient Dignity Inventory (PDI), which is used to assess dignity in PwD [16], was not examined in their study [12]. The domains assessed in the PDI might however, contribute differently to the overall perception of dignity, and these associations may be gender sensitive.

#### Specifics of dignity in men and women with dementia

The differences in dignity between men and women with dementia are worth addressing if only because dementia itself differs between both groups [17]. Women are more likely to suffer from dementia than men, and their disease usually progresses more rapidly [18]. The differences in a prevalence, course or outcomes of dementia might be associated with factors relating to sex (biological attributes of physical body of male or female [19], such as different physiological factors, which might be associated with dementia [20]) and gender (complex patterns of social roles, identities, norms, values and behaviours of male, female and gender-diverse persons [19], such as different roles in society or education of men and women [20]. Although, no previous research has been done to clarify the association between gender and perceptions of dignity in PwD, research findings in other groups of older patients can provide us some insight. In a previous study in individuals at the end of life [21], it was found that female gender, have influence on reduced sense of dignity. Women considered that psychological factors (e.g., the inability to think clearly, feelings of depression and anxiety) and social factors (a person feeling that they are a burden on others, a sense of loss of privacy) had a greater impact on their dignity than their health status. In comparison, in a study of nursing home residents [22], some physical and/or long-term care items were rated as more likely to impact negatively upon their dignity by male than by female respondents. In contrast, gender did not show a significant association with dignity in a study of patients with terminal cancer [23]. Therefore, the relationship between perceived dignity and gender is unclear in older-adult patients. Since research is limited in the area of dignity of PwD, and the gender perspective must be taken into account in order to fully understand the factors related to the perception of dignity [4, 24, 25], the goal of the present study was to discover any factors that affect dignity differently in men and women.

### Cultural specifics of the Czech men and women

In the Czech Republic there are differences in family, societal and employment life, average level of education, and representation in decision-making (such as political representation or managerial positions) between men and women [26].

In terms of family life, the Czech Republic supports maternal and parental leave for child-care up to 4 years of age. There is limited access to day-care facilities for children under three years. Almost exclusively, it is the mother, who stays at home with the children. Long career breaks affect women’s position in the labour market [27], and deepen the employment and pay gap between men and women, which is among the highest in Europe [26]. Taking the impact of lower pensions [28] and longer life expectancy together, older Czech women experience poverty and social exclusion much more than men.

In later life Czechs often continue to work and also take on the responsibility of caring for their aging relatives, as trust in institutional care is not very strong in the post-socialist Czech Republic [29]. The family caregivers are mostly women [30]. Men also look after their parents, but not as frequently, nor as intensely, and when the older-adults’ needs grow the men’s involvement in care decreases [30]. There is limited research on men’s gender roles in Czech society. In a recent qualitative study conducted with grandfathers [31], the participants indicated and endorsed differences in gender roles between men and women. Men perceived the their role to be as a breadwinner during productive lives and as grandparents, they taught their grandchildren masculine roles [31], whereas women consider caring for their relatives as their gender role, their responsibility that constitutes an important part of their personal identity, and duty [29]. In a society with traditional division of gender roles, men do not often talk about their problems, and they avoid requesting help [32]. This might be related to the idea of male identity being based on self-reliance, physical and mental strength, which, in older Czech men, might be reinforced by experience of compulsory military service [33]. These specifics may be important in understanding how Czech men and women differently perceive their dignity.

### The present study

To our knowledge, there are no published studies of whether men and women with dementia differ in their experience of dignity. To examine factors that could be related to dignity in men and women with dementia living in the community, the present study focused on variables that have previously been suggested to relate to dignity in PwD. These were pain, depression, attitudes to aging, self-sufficiency in ADL [12], sociodemographic characteristics (such as age, education), and also the characteristics of social involvement of the participant (e.g. living arrangements, involvement in social activities). We also included visual and hearing impairments and physical performance as independent variables. Both are related to health outcomes [34–36], quality of life [37, 38] and self-sufficiency in ADL [37] and thus, we hypothesize a link between these variables and dignity. We expect to find different factors contributing to dignity and its domains in men and women with dementia. Understanding which factors affect self-perceptions of dignity in PwD can further pave the way toward more effective dignity-conserving, community-based care.

## Methods

### Participants

The research sample consisted of PwD living in the Czech Republic, and the research was conducted in their native Czech language. We used a non-probability sampling method combining criterion and convenience sampling. Firstly, we defined inclusion and exclusion criteria. Secondly, we approached the criterion fitting patients on the basis of their accessibility and availability. The inclusion criteria were as follows: (1) age ≥ 60 years; (2) living in the community rather that in residential care (3) diagnosed with any type of dementia in an early stage (diagnosis according to the International Statistical Classification of Diseases and Related Health Problems [ICD] -10 Version: 2019: F00, F01-F03; Mini-Mental State Examination [MMSE] with a score of 20–25 points). The exclusion criteria for all respondents were as follows: (1) permanent institutional care; (2) complete immobility; (3) a severe psychological disorder (schizophrenia, bipolar affective disorder); (4) a severe sensory disability (blind, deaf); and (5) terminal stage of an oncological or non-oncological disease. We were interested in the community-dwelling patients because this group is less studied than the older PwD living in institutions. For this group, it could also be assumed that their difficulties with dignity would be related to the nature of the illness rather than to the situation of living in the institution.

The respondents were approached through neurological and geriatric outpatient departments, placed in different parts of the Czech Republic, where they were being treated for dementia, so it was ensured that the person was indeed diagnosed with the illness. During their regular check-up, they were offered the opportunity to participate in the study and it was explained what questionnaires they would fill out. None of the respondents refused and all signed informed consent before inclusion in the study. They were competent and independent in their decision. Researchers explained to the participants how to complete the questionnaires. The participants filled out the tools by themselves or with help of the researcher, as a structured interview, if it was preferred. The data was collected from June 2020 to June 2021 in three regions of the Czech Republic.

### Measures

The cross-sectional study was conducted as part of the longitudinal study “Changes in the perception of personal dignity over the course of dementia” (registered in Clinical Trials.gov.; No. NCT04443621). Independent variables were the sociodemographic characteristics of the participants and characteristics related to general physical and mental health (pain, physical performance, sensory impairments, and depression), attitudes to aging and self-sufficiency in ADL. Dignity and its domains were the dependent variables in our study.

#### Independent variables

Sociodemographic and social involvement information was gathered during structured interviews. All variables, except for age, were dichotomized. An interviewer asked about participants’ level of education (dichotomized as lower [elementary school, vocational] and higher [secondary school, university] education), and their living arrangements (dichotomized as living alone, living with others). Regarding social involvement, participants were asked when they participated in a social activity for the last time (more than 30 days ago or 30 or less days before the interview), how long it had been from a friend or a relative visited them (more than 30 days ago or 30 or less days before the interview), how long it was from the last email or telephone contact with friends or relatives (more than 7 days or 7 or less days before the interview), and how many hours a day does a participant spend alone (dichotomized as whether the participant spent more than 8 h 8 or less hours alone daily.

#### Pain

Perceived pain was graphically assessed on the Horizontal Visual Analogue Scale (HVAS), which consists of a continuous10-cm line at which the patient records the level of subjectively perceived pain (no pain to extreme pain) [39, 40]. HVAS can be successfully used in most PwD [39].

#### Physical performance

To assess physical performance, we used the Short Physical Performance Battery (*SPPB,* [41]; Czech version [42]). The SPPB includes assessments of balance, gait speed, and chair rises, that can be administered easily and quickly. The total score ranges from 0 to 12. Higher scores indicate a higher physical performance. SPPB total score ≤ 6 points is rated as a frail older adult [41].

#### Self-sufficiency in activities of daily living

Participants’ self-sufficiency in ADL was assessed by the Bristol Activities of Daily Living Scale (BADLS, [43]; Czech version [44]) that covers basic and instrumental ADL, from completely independent to completely dependent. The questionnaire is completed by the carer of the PwD, who evaluates the performance of 20 activities in the life of the patient [43]. The overall BADLS score ranges from 0 (completely independent) to 60 points (completely dependent). In the Czech version (BADSL-CZ), the score is also converted into percentages representing the range of self-sufficiency (0–100%), where 100% means the complete self-sufficiency in ADL of the person being evaluated [44].

#### Visual and hearing impairments

Participants’ visual and hearing impediments were estimated by a clinician using a screening test of visual acuity for distance (optotype) and near vision, and a subjective hearing examination (speech testing). They were dichotomized as no/minimum or medium/severe impairment.

#### Depression

We estimated depression status by the Geriatric Depression Scale (GDS-15, [45]; Czech version [46]) containing 15 self-assessment items. The total GDS-15 score ranges from 0 to 15 points. The higher the total score, the greater the severity of depression (0–5 points are considered normal, more than 5 points indicates depression). GDS-15 has 92% sensitivity and 89% specificity when evaluated according to diagnostic criteria and distinguishes depressed patients from highly correlated non-depressive adults (*r* = 0.84, *p* < 0.001; [45]). It is a valid and reliable tool for screening for depression that can also be used in PwD [47].

#### Attitude to Aging

We used the Attitude to Aging Questionnaire (AAQ, [48]; Czech version [49]), which consists of 24 items that are divided into three domains: Psychosocial Loss, Physical Change, and Psychological Growth (IRT [Item Response Theory] equivalents of Cronbach’s values for these domains: 0.81, 0.81, and 0.74, respectively). Each domain score is from 8 to 40 points. The total AAQ score ranges from 24 to 120 points. Higher scores indicate more positive attitudes to aging [48]. The applicability of this scale in PwD was confirmed by a previous study [50].

### Dependent variables

#### Dignity and its domains

Dignity was estimated by *The Patient Dignity Inventory* (PDI, [16]; Czech version PDI-CZ, [51]), a 25-item questionnaire, which concentrates on understanding the problems connected with patient dignity. The total score of the questionnaire ranges from 25 to 125 and it is a sum of individual items. A higher score indicates a greater threat to dignity [16]. The scores of the PDI may be divided into four categories: ‘mild’ (25–49 points); ‘moderate’ (50–74 points); ‘severe’ (75–99 points); and ‘very severe’ (100–125 points) [52]. The suitability of the PDI for use for PwD has been demonstrated earlier [53]. The Czech version, PDI-CZ, is based on items divided into four subscales following a factor analysis (‘Loss of purpose of life’; ‘Loss of autonomy’; ‘Loss of confidence’; and ‘Loss of social support’ [internal consistencies range, Cronbach’s α 0.58–0.90]; [51]). The Czech version was validated in PwD by a previous study [12] We examined the total PDI-CZ [51] as the overall experience of dignity, and the four domains of dignity as dependent variables in our study. The domain ‘Loss of purpose’ consists of 13 items and it is related to life purpose in relation to illness, self–appraisal, and future. ‘Loss of autonomy’ items are connected with self-care, dependency, and reactions from the environment. The domain of ‘Loss of confidence’ is related to the mental and existential insecurities. Questions relate to inability to think clearly, feelings of depression or anxiety, and spiritual concerns. The domain of ‘Loss of social support’ consists of three items mapping respondents feelings of being supported by friends, family or health care providers and being treated with respect [51].

### S*tatistical analysis*

Ratio variables were presented using average, standard deviation, and minimum and maximum values. Discrete variables were described using absolute and relative frequencies. The differences between the two independent selections for discrete data were verified using the Accurate Fisher Test. The differences between the two independent selections in quantitative data were calculated using a two-sample t-test. The Mann–Whitney *U* test was used for ordinal quantities. The ANCOVA method was used to distinguish the relationship between gender and the physical performance of PwD from the influence of age. All tests were carried out at the level of statistical significance *p* = 0.05.

The multivariate linear regression assessed the link of sociodemographic characteristics, sensory impairments, pain, depression, physical performance, levels of self-sufficiency in ADL, and attitudes to aging with the perception of dignity, individually for men and women. Prior to the analysis, the regression diagnostics of linearity, multicollinearity, and homogeneity, as well as the normality and independence of residues, were performed. The model was built using the ENTER method. IBM SPSS Statistics for Windows, Version 23.0 (IBM Corp., Armonk, NY, USA) was used for statistical processing.

## Results

The sample consisted of 316 PwD (119 men and 197 women). Their demographic, social involvement, clinical and psychological characteristics are presented in Table 1. The men were significantly younger, had higher education, and were more likely to suffer from hearing impairment. The women lived alone more often and had poorer physical performance. Further, 52.3% of the women and 33.6% of the men were classified as frail older adults (SPPB total score ≤ 6 points; *p* = 0.0005). Given the correlation of physical performance with age found in this study, the relationship between gender and physical performance was adjusted for the effect of age using ANCOVA. Even after performing ANCOVA, the difference between men and women in physical performance remained significant (*p* = 0.009). No statistically significant differences were found between men and women in perception of dignity (PDI-CZ). The total PDI-CZ score (41.9 vs 43.1, *p* = 0.493) represented the category of “mild problems” [52].

**Table 1:** Sociodemographic and clinical characteristics of the respondents

Characteristic	Categories	Male (*N* = 119)	Female (*N* = 197)	*p*-value
**Age** Mean; SD (range)		80.8; 7.7 (60–97)	83.0; 7.2 (64–97)	.011^a^
**Education** N (%)	Elementary	11 (9.2)	62 (31.5)	.0004^b^
	Vocational	55 (46.2)	61 (31.0)	
	Secondary	35 (29.4)	67 (34.0)	
	Tertiary	18 (15.1)	7 (3.6)	
**Social involvement**				
With whom the older adult lives N(%)	Alone	20 (16.8)	95 (48.2)	< .0001^b^
	With partner	87 (73.1)	69 (35.0)	
	With others	12 (10.1)	33 (16.8)	
Participation in social activities N(%)	> 30 days ago	38 (32,0)	78 (39.6)	.655^**b**^
	< 30 days ago	70 (58.8)	101 (51.3)	
	Cannot be determined*	11(9.2)	18 (9.1)	
Visit of relatives/friends N(%)	> 30 days ago	9 (7.6)	12 (6.1)	.161^**b**^
	< 30 days ago	110 (92.4)	185 (93.9)	
Contact with relatives/friends (phone, email) N(%)	> 7 days ago	18 (15.1)	30 (15.2)	.891^**b**^
	≤ 7 days ago	101 (84.9)	167 (84.8)	
Time spent alone daily N (%)	≥ 8 h	29 (24.4)	70 (35.5)	.217^**b**^
	< 8 h	90 (75.6)	127 (64.5)	
**Clinical characteristics**				
Hearing Impairment N (%)	No/Minimum	97 (81.5)	175 (88.8)	.024
	Medium/Severe	22 (18.5)	22 (11.2)	
Visual Impairment N (%)	No/Minimum	104 (87.4)	160 (81.2)	.098
	Medium/Severe	15 (12.6)	37 (18.8)	
Depression (GDS-15 score [Mean; SD]))		4.9; 3.8	5.1; 3.9	.651^a^
Self-Sufficiency in ADL (BADLS-CZ % [Mean; SD])		77.3; 20.5	76.7; 18.8	.788^a^
Pain (HVAS score [Mean; SD])		2.1; 2.6	2.5; 2.7	.253
Physical Performance (SPPB total score [Mean; SD])		7.1; 3.7	5.6; 3.8	.001
**Psychological characteristics**				
Dignity (PDI-CZ [Mean; SD])		41.9; 15.5	43.1; 15.5	.493
Domains Loss of Purpose of Life		22.5; 8.8	23.4; 9.1	.378
Loss of Autonomy		8.7; 3.5	8.9; 3.9	.772
Loss of Confidence		6.8; 2.8	7.2; 2.8	.288
Loss of Social support		3.8; 1.8	3.7; 1.3	.342
Attitude to Aging (AAQ total score [Mean; SD])		73.7; 13.3	72.6; 10.0	.428

* Category cannot be determined, excluded from the statistical comparison

^a ^Independent samples *t-*test

^b ^Mann-Whitney U-test

^c ^Fisher exact test

### Contributors to dignity in women with dementia

None of the sociodemographic or social involvement characteristics showed a significant association with dependent variables in women (Table 2). From physical health related characteristics, pain contributed to the perception of dignity in the domains of ‘Loss of Purpose of Life’ and ‘Loss of Autonomy’ and the overall PDI-CZ (ß = 0.722, *p* = 0.027) in women. The greater the pain the women reported, the worse they evaluated their dignity. Physical performance or sensory impairments did not contribute to any dependent variable in women. In women, depression contributed to the overall dignity (PDI-CZ) and in all domains of PDI-CZ (Table 2). The higher the level of depression, the worse the women rated their dignity. Attitude to aging was related to the overall PDI-CZ and the domains of ‘Loss of Purpose of Life’, ‘Loss of Autonomy’, and ‘Loss of Confidence’ of the PDI-CZ in women (Table 2). Women, who had more positive attitude to aging rated their dignity better.

**Table 2:** Linear regression model – women

	**Domains of PDI-CZ**									**Dignity (PDI-CZ)**	
Variables	Loss of Purpose of Life		Loss of Autonomy			Loss of Confidence		Loss of Social Support			
	*ß*	*p*	*ß*	*p*	*ß*		*p*	*ß*	*p*	*ß*	*p*
Constant	29.333	.021	15.078	.006	6.683		.076	-0.646	.769	50.448	.015
Age	-0.028	.737	0.003	.923	-0.001		.980	0.012	.403	-0.013	.923
Education^a^	0.386	.726	0.188	.688	-0.254		.437	0.021	.911	0.342	.849
Living Arrangements^b^	0.968	.366	0.095	.835	0.570		.073	0.290	.121	1.923	.271
Participation in Social Activities^c^	1.996	.066	0.765	.098	0.518		.108	0.384	.053	3.663	.059
Visiting Friends^d^	-3.192	.234	-1.993	.081	-0.723		.362	0.144	.757	-5.764	.188
Phone/email Contacts^e^	-1.183	.475	-0.683	.333	-0.281		.567	-0.174	.547	-2.321	.391
Hearing Impairment	0.224	.888	0.773	.253	0.424		.367	0.404	.145	1.825	.481
Visual Impairment	-0.619	.642	-0.045	.936	-0.144		.716	0.137	.557	-0.672	.757
Pain (HVAS)	0.483	.016^*^	0.217	.011^*^	0.072		.222	-0.049	.154	0.722	.027^*^
Physical Performance (SPPB)	0.086	.654	0.010	.903	0.108		.058	0.016	.635	0.220	.482
Depression (GDS-15)	1.166	< .0001^***^	0.277	.0001^***^	0.455		< .0001^***^	0.155	< .0001^***^	2.053	< .0001^***^
Attitude to Aging (AAQ)	-0.205	.002^**^	-0.061	.028^*^	-0.041		.034^*^	-0.015	.180	-0.321	.003^**^
Self-Sufficiency in ADL (BADLS-CZ)	-0.122	.002^**^	-0.097	< .0001^***^	-0.030		.013^*^	0.0004	.950	-0.248	.0002^***^
R^2^/adjusted R^2^	.564/.526		.547/.508		.575/0.539			.304/.244		.600/.565	
Durbin-Watson Test/VIF	1.763/1.523		2.006/1.523		1.856/1.523			1.941/1.523		1.792/1.523	

^*^p < 0.05; ^**^p < 0.01; ^***^p < 0.001

^a ^category dichotomized by the level of education into lower education (elementary, vocational) vs. higher (secondary, tertiary)

^b ^category dichotomized by the living arrangement into living alone vs. not alone (with partner or other people)

^c ^category dichotomized by the last time participated at social activity into > 30 days ago vs. 30 or less days ago

^d ^category dichotomized by the last time visit of a friend/relative into > 30 days ago vs. 30 or less days ago

^e ^category dichotomized by the last time of the phone/email contact with a friend/relative into > 7 days ago vs. 7 or less days ago. For each dichotomous variable, the first one listed was the reference category

Self-sufficiency in ADL contributed to the overall PDI-CZ and the domains of ‘Loss of Purpose of Life’, ‘Loss of Autonomy’, and ‘Loss of Confidence’. Women who had a higher self-sufficiency in ADL perceived their dignity as better. In women, the determination coefficient R^2^ was highest for the model for the dependent variable “total PDI-CZ” – overall dignity (explained 57.0% of variance), where pain, depression, attitude to aging and self-sufficiency in ADL where significant contributors to the dependent variable. The lowest coefficient R^2^ was for the model for the dependent variable ‘Loss of social support’ (24.4% of explained variance), where depression was the only significant contributor of the dependent variable (Table 2).

### Contributors to dignity in men with dementia

In men, none of the sociodemographic or social inclusion characteristics showed a significant association with dependent variables (Table 3). From health-related characteristics, pain and sensory impairment contributed to the dependent variables. Pain was a contributor to the overall PDI-CZ (*ß* = 1. 464, *p* < 0.0001) and in all the domains of PDI-CZ except ‘Loss of Social Support’ (Table 3). The greater pain the men perceived, the worse they evaluated their dignity. Medium or severe visual impairment had a negative effect on the men’s experience of dignity in the domains of ‘Loss of Autonomy’ and ‘Loss of Confidence’ and in the overall PDI-CZ (Table 3). Physical performance and hearing impairment were not associated with any of the dependent variables.

**Table 3:** Linear regression model – men

	**Domains of PDI-CZ**								**Dignity (PDI-CZ)**	
Variables	Loss of Purpose of Life		Loss of Autonomy		Loss of Confidence		Loss of Social Support			
	*ß*	*p*	*ß*	*p*	*ß*	*p*	*ß*	*p*	*ß*	*p*
Constant	33.110	.008	6.611	.227	8.816	.034	8.627	.009	57.164	.006
Age	0.029	.665	0.021	.494	0.000	.997	-0.022	.215	0.027	.808
Education^a^	0.878	.411	0.495	.300	0.421	.242	-0.087	.759	1.707	.340
Living Arrangements^b^	-0.161	.903	-0.140	.812	0.302	.497	0.060	.864	0.061	.978
Participation in Social Activities^c^	0.239	.826	0.171	.725	0.144	.695	0.237	.414	0.791	.664
Visiting Friends^d^	-0.429	.882	0.865	.503	-0.493	.613	0.901	.243	0.845	.861
Phone/email Contacts^e^	-1.328	.461	-0.239	.766	-0.572	.345	-0.915	.058	-3.055	.311
Hearing Impairment	-0.120	.935	-0.339	.607	-0.244	.622	0.222	.571	-0.481	.845
Visual Impairment	2.103	.165	1.898	.006^**^	1.025	.046^*^	0.337	.402	5.364	.036^*^
Pain (HVAS)	0.848	< .0001^***^	0.312	.001^**^	0.253	.0003^***^	0.052	.325	1.464	< .0001^***^
Physical Performance (SPPB)	-0.180	.342	-0.111	.190	-0.013	.840	-0.070	.167	-0.373	.239
Depression (GDS-15)	1.269	< .0001^***^	0.339	.0002^**^	0.474	< .0001^***^	0.137	.009^**^	2.220	< .0001^***^
Attitude to Aging (AAQ)	-0.169	.018^*^	-0.040	.207	-0.027	.253	-0.033	.080	-0.269	.025^*^
Self-Sufficiency in ADL (BADLS-CZ)	-0.006	.876	-0.048	.006^**^	0.001	.908	0.014	.181	-0.039	.543
R^2^/adjusted R^2^	.747/.709		.686/.639		.719/.676		.441/.357		.773/.739	
Durbin-Watson test/VIF	1.934/1.746		1.936/1.746		1.845/1.746		2.288/1.746		1.946/1.746	

^*^p < .05; ^**^p < .01; ^***^p < .001

^a ^category dichotomized by the level of education into lower education (elementary, vocational) vs. higher (secondary, tertiary)

^b ^category dichotomized by the living arrangement into living alone vs. not alone (with partner or other people)

^c ^category dichotomized by the last time participated at social activity into > 30 days ago vs. 30 or less days ago

^d ^category dichotomized by the last time visit of a friend/relative into > 30 days ago vs. 30 or less days ago

^e ^category dichotomized by the last time of the phone/email contact with a friend/relative into > 7 days ago vs. 7 or less days ago. For each dichotomous variable, the first one listed was the reference category

In men, depression was a contributor to the overall dignity—PDI-CZ (*ß* = 2.220; *p* < 0.0001) and to all domains of dignity (Table 3). The higher the level of depression, the worse the men rated their dignity.

Attitude to aging influenced the overall PDI-CZ (*ß* = –0.269, *p* = 0.025) and the domain of ‘Loss of Purpose of Life’ (Table 3). If men had a better attitude to aging, they evaluated their dignity better.

Self-sufficiency in ADL was associated only with the domain of ‘Loss of Autonomy’ in men (Table 3). If men had better self-sufficiency in ADL, they evaluated their dignity better. The coefficient of determination *R*^2^ was the highest for the model for the dependent variable “Total PDI-CZ” – overall dignity (73.9% of explained variance) with visual impairment, pain, depression, and attitude to aging being significant contributors of the dependent variable. The coefficient of determination *R*^2^ was lowest for the model for the dependent variable ‘Loss of Social Support’ (35.7% of explained variance) (Table 3), with depression being the only significant contributor of the dependent variable.

## Discussion

This study focused on dignity, and its associated domains, in men and women in the early stages of dementia. The results are in line with previous findings that suggested PwD experienced reduced dignity or a threat to it [3, 5, 10, 12], regardless of gender. However, both women and men exhibited only minor problems in dignity. One of the explanations is that the participants lived in their own homes, which allowed them to keep their social role, and control their life. This assumption is supported by a previous qualitative study that found only minor issues with dignity described by PwD living in their homes [3]. Although it seems that continuing to live within the community may lead to better preservation of dignity, it has previously been found that even in institutional care dignity may be preserved when older adults trust their professional caregivers, have control over their decision making, and feel to be a part of a social network [54]. Further study is needed to compare perceptions of dignity in PwD living at home with those in institutional care. From a gender perspective, it is possible that men did not want to admit their feelings of reduced dignity because this would jeopardize their masculine identity [33]. Women might downplay their problems because they do not want to worry others – perceiving that they are the ones who are supposed to take care of their loved ones. Their lifelong focus on caring for children and other relatives could also distract them from paying attention to their own difficulties and needs, including the area of dignity. It is also possible that PwD reported minor dignity issues because they perceived other issues, such as physical symptoms and needs, as more pressing from their viewpoint.

Pain, depression, and the attitude to aging were common contributors to the overall perception of dignity (PDI-CZ) in both women and men with dementia. The finding that pain predicted a diminished perception of dignity was consistent with a previous study in terminally ill patients [6], in which, the experience of pain was associated with loss of dignity probably by affecting the individual’s competence, autonomy, and sense of self-worth. Lowered sense of competence or self-worth might be closely related to the domain ‘Loss of Confidence’, which was associated with pain in men. Possibly, the perceived pain threatens men’s identity by reminding them of their weakening physical strength, which result in loss of confidence. Our results showed that pain was also correlated with ‘Loss of Purpose’ and ‘Loss of Autonomy’ in men and women. It may be that the reduced ability to perform both fulfilling and routine everyday activities is the mechanism by which pain reduces dignity in these domains. Family and professional caregivers should be informed that pain is often under-recognized and undertreated in PwD and they should be taught how to recognize its symptoms [55]. Alleviating pain can have impact on overall wellbeing of PwD including their improved perception of dignity.

Depression was the only variable associated with both overall dignity and also with all of its domains in both genders. Higher rates of depression predicted lower perception of dignity. A link between dignity and depression has also been found in terminally ill patients [6]. Depression can deepen negative experiences in the areas of emotional or physical dependence on others, feelings of shame, and feelings of being a burden. It also comes with a decreased sense of self-worth and self-confidence and can thus lead to a reduced perception of the persons dignity [56]. Depression can promote negative experiences leading to a decrease in dignity and also in overall quality of life [5]. Hence, screening for the timely diagnosis of depression in PwD and its effective treatment should be carried out not only to improve mental health, but also to protect dignity.

A positive attitude to aging contributed to improved perception of dignity in both women and men with dementia. The relationship between these variables has already been pointed out, although that study considered dignity as a predictor of attitude to aging [12]. In addition to the overall PDI-CZ in women, the attitude to aging also contributed to the ‘Loss of Purpose of Life’, ‘Loss of Autonomy’, and ‘Loss of Confidence’; in men, it was only associated with the domain of ‘Loss of Purpose of Life’. The reason why attitudes to aging are related to more dimensions of dignity for women than for men may be due to the fact that aging is generally a more salient issue for women [57]. They have more negative attitudes towards aging than men and have more concerns about old age [57, 58]. Attitude to aging in women with dementia is associated with experience of loneliness, social exclusion and gradual loss of physical self-sufficiency [12], which are closely related to the domains of dignity.

A previous study suggested that a positive attitude to aging and, by extension, the perception of old age as a meaningful stage of life, is a factor that may help to preserve dignity [3]. This found that PwD, who believe that their lives still make sense are better at maintaining a sense of personal dignity. Another study found a link between a positive attitude to life and the perception of dignity in nursing home residents [22]. Future research should focus on the domains of the AAQ (Attitude to Aging Questionnaire) in relation to dignity and its domains to better understand the differences between men and women. Psychosocial interventions that could improve attitude to aging might be beneficial. They could include socialization activities, counselling, or reminiscence therapy, which were shown to improve attitudes to aging in PwD in previous research [59]. Reminiscence therapy could be focused on the individual’s life-projects, personal skills, values, and former meaningful work as these are important sources of self-worth and self-esteem [9].

In women, self-sufficiency in ADL was associated with overall dignity and with all four domains of the PDI-CZ. In men, self-sufficiency in ADL contributed only to the PDI-CZ domain of ‘Loss of Autonomy’. A link between self-sufficiency in ADL and dignity has been found also in previous qualitative [60–62] and quantitative [6, 12] studies. Maintaining self-sufficiency in ADL, and therefore functional autonomy, is considered to be one of the central conditions of dignity [61], and the idea of the loss of self-sufficiency is one of the major concerns in relation to old age [60]. The association between the self-sufficiency in ADL and the ‘Loss of Autonomy’, which was found in both genders, was expected, since both variables are relate to dependency and autonomy [43, 51]. We hypothesize that the decreased self-sufficiency in ADL is, especially in women, associated with lower perception of dignity because it may limit their customary gender roles. For example, the sudden change of women’s role from a care provider to a care recipient, may strongly challenge their gender identity, and consequently their dignity. If self-sufficiency is reduced, their ability to care for others) is probably limited. Because of relatively reduced access to affordable childcare facilities in the Czech Republic [26], older women often take care of their grandchildren and build a close relationship with them. Therefore, being a grandmother is important to women’s identity and self-worth, and gives them a clear purpose for life in old age. Thus, we consider that the relationship between experienced dignity and regular contact with grandchildren would be worth further study. Reduced self-sufficiency in ADL might also be a barrier for caring for one’s own home. This task is not only more commonly performed by women but is important to the identity of women with dementia, affecting their sense of competence, and self-worth [13].

From a practical point of view, this study demonstrated that activities aimed at maintaining the patient’s autonomy or strengthening competencies that had not yet been affected by the illness, might be important to supporting the perception of dignity in PwD. With regard to gender, community caregivers should encourage activities related to men’s or women’s roles. For example, they should support women’s need for caring, show them how they are still capable of taking care of others, and that they are needed by their social network. It can be done through performing activities together but also, in the case of physical issues, the caregivers can ask for advice, (e.g. an easy question “how to bake a cake” can make the person feel needed). Future studies should focus on factors in both men and women that may affect the relationship between the perception of dignity and self-sufficiency.

In men, overall dignity, ‘Loss of Autonomy’, and ‘Loss of Confidence’, were also associated with a visual impairment. No study has been reported that supports this link in PwD. Because there was no significant difference in the degree of visual impairment of men and women in the study population, it is possible that men perceive its’ impact on their everyday life differently than women, in ways that have implications for their dignity. Theoretically, visual impairment can prevent men from activities typical of their gender, and male identity (e.g., driving a car, playing games, or solving crosswords). Regular eye tests should be performed in PwD, as they help optimal correction of visual impairments, which might have a positive impact on the perception of dignity. Patients and their caregivers should be educated about dealing with visual impairments (information on screen readers, interior adjustments etc.). They could be offered assistive technology or equipment, and rehabilitation for visually impaired if needed.

The results of the present study suggested that involvement in social life was not related to the perception of dignity in PwD. However, a previous qualitative study, which included PwD living in their own home [3], suggested that how they experienced their dignity was related to their social environment. It is possible that women and men with dementia in the present study did not experience much limitation of their social life as compared to their previous lives or maintained as much social contact as they wished. This possibility was supported by the fact that the participants reported the fewest difficulties in the ‘Loss of Social Support’ compared with the other PDI-CZ domains. Nevertheless, we believe the social environment and its associations with dignity in PwD deserves deeper attention in future studies. In our study, we observed the frequency of contacts with others (visits or phone/email), or participation in social activity and, in terms of living arrangements, we only recorded whether the respondent lived alone or not. It is possible, that the frequency of contacts alone is not as important to the perception of dignity as other aspects of social involvement or the environment, such as the quality of the relationship, satisfaction with the relationship, contact with grandchildren or marital status. Future study should focus on these aspects in more detail.

### Limitations

The present study was novel in providing valuable insights into the issue of dignity in PwD in terms of gender. However, it is necessary to mention some of the limitations of the study that should be considered when interpreting the results. The results could not be generalized to the entire population of PwD because it includes only individuals living in their home environment and at an early stage of the disease, and makes no distinction between the different types of dementia. The current study is also of a cross-sectional nature and thus we cannot assess causality. Further studies should be longitudinal to clarify causal relationships. This study did not look at other potential factors that could contribute to the dignity in PwD including comorbidities, psychiatric treatment, emotional regulation, and distress or anxiety. For example, we did not control for whether participants had been diagnosed with depression before they were diagnosed with dementia. An important implication for future studies would be looking at clusters of the various types of dementia, as they manifest different behavioural and psychological symptoms [63], which may affect the subjective experiences and also perception of dignity in PwD. The relationships found might also have been influenced by the cultural context in which the study was conducted.

## Conclusions

The results of this study suggest that personal perceptions of dignity were associated with attitude to aging, depression, and pain in both men and women. In women, a reduced perception of dignity was also associated with reduced self-sufficiency in ADL. In men reduced perception of dignity was associated with visual impairment. Physical performance and the aspects of the social involvement investigated were not associated with perceptions of dignity for either men or the women. The study showed that dignity could be compromised in PwD who lived outside an institutional environment and that it was related not only to health factors but also to psychological variables such as attitudes to aging or depression in men and women. The research findings can be used in the provision of medical, psychosocial, and nursing care to PwD.

## Data Availability

The datasets supporting the conclusions of this article are available from the corresponding author on a reasonable request.

## Abbreviations

AAQ: Attitude to Aging Questionnaire
ADL: Activities of Daily Living
BADLS: The Bristol Activities of Daily Living Scale
BADSL-CZ: The Czech version of the Bristol Activities of Daily Living Scale
GDS-15: The Geriatric Depression Scale
HVAS: The Horizontal Visual Analogue Scale
PDI: The Patient Dignity Inventory
PDI-CZ: The Czech version of the Patient Dignity Inventory
PwD: People with Dementia
SPPB: The Short Physical Performance Battery
